# Loss of the histone chaperone UNC-85/ASF1 inhibits the epigenome-mediated longevity and modulates the activity of one-carbon metabolism

**DOI:** 10.1016/j.cstres.2024.04.003

**Published:** 2024-04-10

**Authors:** Bideep Shrestha, Anni I. Nieminen, Olli Matilainen

**Affiliations:** 1 The Molecular and Integrative Biosciences Research Programme, Faculty of Biological and Environmental Sciences, University of Helsinki, Helsinki, Finland; 2 FIMM Metabolomics Unit, Institute for Molecular Medicine Finland, University of Helsinki, Helsinki, Finland

**Keywords:** ASF-1, UNC-85, Lifespan, One-carbon metabolism, *C. elegans*

## Abstract

Histone H3/H4 chaperone anti-silencing function 1 (ASF1) is a conserved factor mediating nucleosomal assembly and disassembly, playing crucial roles in processes such as replication, transcription, and DNA repair. Nevertheless, its involvement in aging has remained unclear. Here, we utilized the model organism *Caenorhabditis elegans* to demonstrate that the loss of UNC-85, the homolog of ASF1, leads to a shortened lifespan in a multicellular organism. Furthermore, we show that UNC-85 is required for epigenome-mediated longevity, as knockdown of the histone H3 lysine K4 methyltransferase *ash-2* does not extend the lifespan of *unc-85* mutants. In this context, we found that the longevity-promoting *ash-2* RNA interference enhances UNC-85 activity by increasing its nuclear localization. Finally, our data indicate that the loss of UNC-85 increases the activity of one-carbon metabolism, and that downregulation of the one-carbon metabolism component *dao-3*/*MTHFD2* partially rescues the short lifespan of *unc-85* mutants. Together, these findings reveal UNC-85/ASF1 as a modulator of the central metabolic pathway and a factor regulating a pro-longevity response, thus shedding light on a mechanism of how nucleosomal maintenance associates with aging.

## Introduction

Nucleosomes, which pack DNA into chromatin, consist of two copies each of the four core histones (H3, H4, H2A, and H2B). These nucleoprotein octamers are highly dynamic complexes, and the turnover of single histones or full nucleosomes enables cells to adapt to different genomic processes.^1^ Histone chaperones are one of the main class of proteins responsible for the dynamic nature of nucleosomes. In addition to nucleosomal histone deposition, these proteins contribute to histone folding, oligomerization, post-translational modification, nuclear import, stability, assembly, and genomic localization, thereby maintaining genomic and epigenetic integrity.^2^ In contrast to the role of histone chaperones in epigenetic maintenance, aging is known to deteriorate the epigenome for example through changes in histone post-translational modifications, altered chromatin remodeling, accumulation of histone variants, and loss of core histones.^4^, ^3^, ^5^, ^6^, ^7^ Since H3 is the most heavily post-translationally modified histone, most of the reported aging-related changes in histone modifications are found in this nucleosome component.^4^, ^3^, ^6^, ^7^ On the other hand, depletion of certain factors regulating the histone H3 post-translational modifications have been shown to promote longevity. For example, data from *Caenorhabditis elegans* demonstrate that inhibition of histone H3 lysine K4 (H3K4) methylation complex,^8^, ^9^ H3K4me3 (H3K4 trimethylation) reader SET-26 (Histone-lysine N-methyltransferase SET-26),^10^ histone H3 lysine K27 (H3K27) demethylase JmjC domain-containing protein (UTX-1),^11^, ^12^ or histone H3 lysine K36 (H3K36) dimethyltransferase Histone-lysine N-methyltransferase SET-18 (SET-18)^13^ extends lifespan. Furthermore, downregulation of H3K4 demethylase Lysine-specific histone demethylase (LSD-1) increases lifespan,^14^ whereas H3K27 demethylase Lysine-specific histone demethylase JMJD-3.1 (JMJD-3.1), and JMJD-3.1 together with another demethylase Lysine-specific demethylase 7 homolog (JMJD-1.2) are required for heat shock response and mitochondrial stress-mediated longevity, respectively.^15^, ^16^ Apart from histone modifications, it has been shown that the accumulation of histone H3 variant H3.3 during aging promotes pro-longevity transcriptional programs and thereby extends lifespan in long-lived *C. elegans* strains.^17^

Based on the mentioned links between histone H3 and aging, it is plausible that histone chaperones responsible for maintaining histone H3 homeostasis play crucial roles in the regulation of longevity. Anti-silencing function 1 (ASF1) is a central histone chaperone facilitating the delivery of newly synthesized histone H3–H4 dimers to nucleosomes, both in a replication-coupled and replication-independent manner.^2^, ^18^ ASF1, expressed in two isoforms in mammals (ASF1A and ASF1B, with ASF1A being the more widely expressed), has been shown to play vital roles in nucleosome assembly and disassembly, replication, transcription, and DNA repair.^18^ In the context of aging, studies in yeast have demonstrated that the depletion of Asf1 leads to a shortened lifespan.^19^ Moreover, it has been shown that histone chaperone Probable histone-binding protein LIN-53 (LIN-53), whose mammalian homolog RbAp48 exchange H3–H4 histones with ASF1 during nucleosome assembly,^20^ is required for a normal lifespan.^21^ However, whether ASF1 regulates lifespan in a multicellular organism remains unexplored, along with the underlying mechanisms. To address these questions, we employed *C. elegans* as a model organism to investigate the role of ASF1 in the aging process.

## Results

### ASF1 homologs are required for normal lifespan in *C. elegans*

**Fig. 1:**
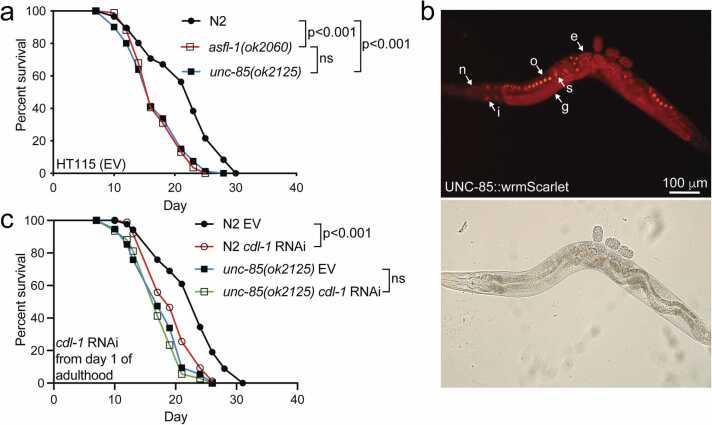
Loss of *C. elegans* ASF1 homologs ASFL-1 and UNC-85 shortens the lifespan. (a) Lifespan of *asfl-1(ok2060)* and *unc-85(ok2125)* mutants compared to wild-type (N2) on *E. coli* HT115 carrying the empty vector (EV). (b) Image of a day 1 adult UNC-85:wrmScarlet-expressing animal (PHX3386). Arrows indicate tissues where UNC-85 is expressed (n, neurons; i, intestine; g, germline, o, oocyte; s, spermatheca; e, embryo). (c) Lifespan of N2 and *unc-85(ok2125)* mutants on *cdl-1* RNAi. Animals were transferred from HT115 (EV) to *cdl-1* RNAi on day 1 of adulthood. Statistical calculations for lifespan experiments were performed using the Cox-proportional hazard regression analysis. Lifespan statistics are reported in Supplementary Material File 1, Table S1. Abbreviations used: ASF1, anti-silencing function 1; EV, empty vector; ns, not significant; RNAi, RNA interference.

*C. elegans* has two ASF1 homologs, Probable histone chaperone ASF-1-like protein (ASFL-1) and probable histone chaperone ASF-1 (UNC-85).^22^ It has been shown earlier that these two histone chaperone homologs have partially overlapping functions in the germline, whereas UNC-85 is mainly responsible for ASF1-related functions in somatic cells.^23^ In this regard, the importance of UNC-85 for somatic cells is underlined by studies showing that the loss of UNC-85 leads to cell division failures during post-embryonic development.^22^, ^24^, ^25^ Despite the reports elucidating the function of UNC-85 and ASFL-1, it is not known how these ASF1 homologs affect lifespan. By utilizing mutant strains carrying *asfl-1(ok2060)* and *unc-85(ok2125)* loss-of-function alleles, we found that both strains have a shortened lifespan compared to the wild-type (N2) (Figure 1(a)), thus mimicking the lifespan phenotype of Asf1-depleted yeast strain.^19^

Tousled-like kinases (TLKs) are evolutionarily conserved serine–threonine kinases that have a role in processes such as DNA replication, DNA repair, and maintenance of chromatin structure and stability.^26^ These kinases are closely associated with ASF1, as TLKs phosphorylate ASF1 to enhance its histone binding and interaction with downstream chaperones.^27^, ^28^ *C. elegans* has one TLK homolog, TLK-1,^29^ which has been shown to phosphorylate UNC-85.^30^ In addition to its link with TLK, another conserved function of ASF1 in multicellular organisms, from *Arabidopsis* and *Drosophila* to mammalian cells, is its essential role in cell replication.^31^, ^32^, ^33^, ^34^ Notably, as mentioned above, UNC-85 has been demonstrated to be required for somatic cell replication in *C. elegans*.^22^, ^24^, ^25^ Finally, as UNC-85 has also been shown to regulate chromatin H3–H4 levels,^35^ we focused on UNC-85 among the two ASF1 homologs due to the above-mentioned conserved ASF1-related functions (being a TLK-1 substrate, regulating cell replication and chromatin H3–H4 levels).

Experiments with a strain carrying the extrachromosomal *unc-85:gfp* transgene have shown that UNC-85 is expressed in replicating cells throughout the development.^22^ To visualize the localization and expression of the endogenous UNC-85, we utilized a strain in which UNC-85 is tagged with the fluorescent wrmScarlet-protein through CRISPR/Cas9-mediated genome editing. This strain revealed that in adult animals, UNC-85 is expressed in both somatic cells and the germline (Figure 1(b)), thus supporting earlier studies describing the function of this histone chaperone in these two cell types. The accumulation of ASF1 upon hydroxyurea (HU)-induced replication stress is an important feature of mammalian ASF1 expression.^31^ To investigate whether UNC-85:wrmScarlet mimics its mammalian homolog, we treated our transgenic animals with HU. Our findings reveal that HU induces the accumulation of UNC-85:wrmScarlet in germ cells and embryos (Figure S1(a)), supporting the use of this strain for studying the expression and localization of ASF1/UNC-85 in *C. elegans*.

Since UNC-85/ASF1 is a central factor in histone chaperone network,^2^ it is plausible that the shortened lifespan observed in *unc-85(ok2125)* mutants is a result of impaired histone deposition into the chromatin. To investigate this, we knocked down *cdl-1*, a homolog of the histone hairpin-binding protein essential for histone synthesis.^36^ Given that *cdl-1* knockdown affects development, we initiated RNA interference (RNAi) treatment on day 1 of adulthood. Interestingly, post-developmental *cdl-1* depletion shortens the lifespan of N2 animals but does not affect the lifespan of *unc-85(ok2125)* mutants (Figure 1(c)). Since the reduction in histone gene expression does not further shorten the lifespan of *unc-85(ok2125)* mutants, these data suggest that the loss of UNC-85 shortens lifespan by impairing histone homeostasis. As previously mentioned, TLKs phosphorylate ASF1 to enhance its activity in maintaining histone homeostasis.^27^, ^28^ Therefore, we explored whether the depletion of TLK-1 affects lifespan. Surprisingly, in our experiments, *tlk-1* knockdown does not influence the lifespan of either N2 or *unc-85(ok2125)* mutants (Figure S1(b)), indicating that UNC-85 may regulate lifespan independently of TLK-1. It is worth noting that these experiments were performed using RNAi, raising the possibility that the knockdown efficiency might not have been sufficient to fully reveal the lifespan-modulating effects of TLK-1.

### UNC-85 is required for the extended lifespan upon knockdown of H3K4 methyltransferase *ash-2*

**Fig. 2:**
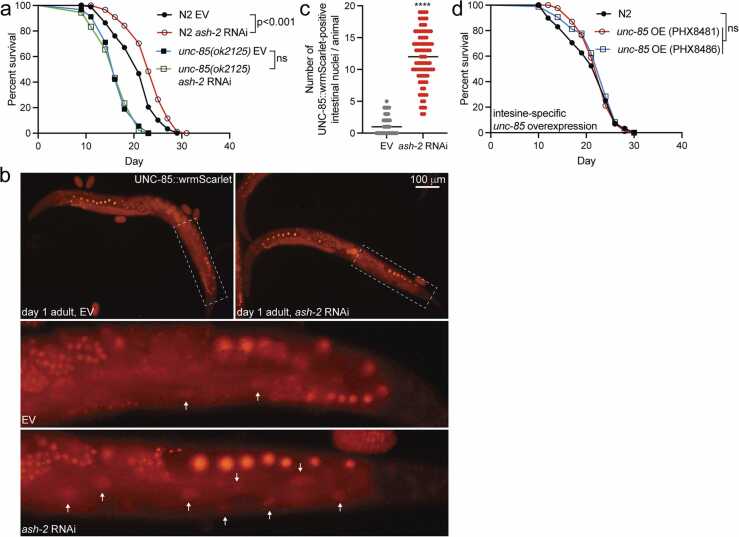
UNC-85 is required for the lifespan extension upon knockdown of H3K4 methyltransferase *ash-2*. (a) Lifespan of N2 and *unc-85(ok2125)* mutants on *ash-2* RNAi. (b) Representative images of UNC-85:wrmScarlet-expressing animals treated with control (EV) or *ash-2* RNAi. The images below, with arrows indicating the nuclear accumulation of UNC-85:wrmScarlet in intestinal cells, are magnifications of the dashed areas. (c) Quantification of UNC-85:wrmScarlet nuclear accumulation in intestinal cells. Each dot represents one animal (n = 81 animals for both conditions, data collected from three independent experiments, ****P < 0.0001, unpaired t-test from the means of the three replicates). (d) Lifespan of N2 and animals with intestine-specific *unc-85* overexpression. Statistical calculations for lifespan experiments were performed using the Cox-proportional hazard regression analysis. Lifespan statistics are reported in Supplementary Material File 1, Table S1. Abbreviations used: EV, empty vector; ns, not significant; RNAi, RNA interference.

As mentioned above, knockdown of components in the ASH-2 H3K4 methyltransferase complex extends the lifespan of *C. elegans*.^8^, ^9^ Experiments with yeast have shown that there is an intriguing link between H3K4me3 and Asf1, as H3K4 methyltransferase Set1-catalyzed H3K4me3 promotes histone gene expression by antagonizing the repressive effect of the HIR/Asf1/Rtt106 complex.^37^ To explore a potential connection between the ASH-2 H3K4 methyltransferase complex and UNC-85, we examined how Set1/Ash2 histone methyltransferase complex subunit ash-2 *(ash-2)* RNAi impacts the lifespan of *unc-85(ok2125)* mutants. Interestingly, while *ash-2* RNAi promotes longevity in N2, it has no effect on the lifespan of *unc-85(ok2125)* mutants (Figure 2(a)). Since *ash-2* RNAi does not affect the lifespan of *unc-85(ok2125)* mutants, we investigated whether UNC-85 deficiency affects *ash-2* knockdown efficiency. For this, we treated N2 and *unc-85(ok2125)* mutants with *ash-2* RNAi from L1 larvae and collected the animals for quantitative RT-PCR (qRT-PCR) analysis as L4 larvae. Although this RNAi regimen results in only approximately a 20% decrease in *ash-2* messenger RNA (mRNA) levels, the effect is similar in both N2 and *unc-85(ok2125)* mutants (Figure S2(a)). To assess the response of *unc-85(ok2125)* mutants to another lifespan-extending RNAi, we downregulated the mitochondrial electron transport chain component *cox-5B* (also known as *cco-1*), a factor previously demonstrated to extend lifespan.^38^ Treatment with *cox-5B* RNAi results in significant lifespan extension in *unc-85(ok2125)* mutants (Figure S2(b)), further suggesting that the loss of UNC-85 does not impact the RNAi pathway and that UNC-85 is not a required factor in all processes promoting longevity. Therefore, the lifespan data (Figure 2(a)) suggest that UNC-85 is essential for the epigenome-mediated longevity induced by ASH-2 inhibition. As the next step, we utilized Western blot to analyze whether *ash-2* RNAi affects the level of H3K4me3 modification differently in N2 and *unc-85(ok2125)* mutants. Surprisingly, we found that *ash-2* knockdown does not affect the H3K4me3 levels in either L4 stage N2 or *unc-85(ok2125)* mutants (Figure S2(c)). Notably, it has been shown earlier that *ash-2* RNAi reduces H3K4me3 levels when the analysis is performed from the lysates of L3-stage animals.^8^ Given the accumulation of the H3K4me3 mark in the germline,^8^ it is plausible that the higher germ cell number in L4 larvae compared to L3 larvae masks the longevity-inducing changes in H3K4me3 levels. In addition to H3K4me3, we also investigated how *unc-85(ok2125)* mutation and *ash-2* RNAi affect the levels of different core histones. We did not observe any consistent and significant differences in these Western blots, except for a decreased level of histone H2B in *ash-2* RNAi-treated N2 compared to the empty vector (EV) and *ash-2* RNAi-treated *unc-85(ok2125)* mutants (Figure S2(c)).

As UNC-85 is required for the *ash-2* RNAi-mediated lifespan extension (Figure 2(a)), we investigated whether *ash-2* knockdown affects the level or localization of UNC-85. For this purpose, we utilized the above-described strain expressing the UNC-85:wrmScarlet fusion protein (Figure 1(b)). Imaging of this strain at day 1 of adulthood revealed that *ash-2* RNAi does not affect the level of UNC-85 (Figure 2(b)). However, we observed that *ash-2* RNAi promotes the nuclear localization of UNC-85 in intestinal cells (Figure 2(b) and (c)). This finding suggests that the knockdown of H3K4 methyltransferase triggers a response resulting in enhanced UNC-85 activity, which may contribute to the observed lifespan extension (Figure 2(a)). Since *ash-2* RNAi enhances the nuclear localization of UNC-85 (Figure 2(b)), we investigated whether intestine-specific overexpression of *unc-85* affects lifespan. For this, we employed two independent strains overexpressing *unc-85* under the intestine-specific *ges-1* promoter. Lifespan experiments with these strains revealed that the elevated UNC-85 level in the intestine does not affect longevity (Figure 2(d)), thus further supporting the hypothesis that the increase in nuclear localization of UNC-85, rather than the upregulation of its total level, is important for extended lifespan. As a drawback, we do not know the localization of overexpressed UNC-85, which limits drawing conclusions based on this experiment. Nevertheless, it is worth noting that Asf1 overexpression has been shown to shorten lifespan in yeast,^19^ underscoring the importance of maintaining a balance in the expression of this histone chaperone.

### The loss of UNC-85 increases the levels of metabolites associated with one-carbon metabolism

**Fig. 3:**
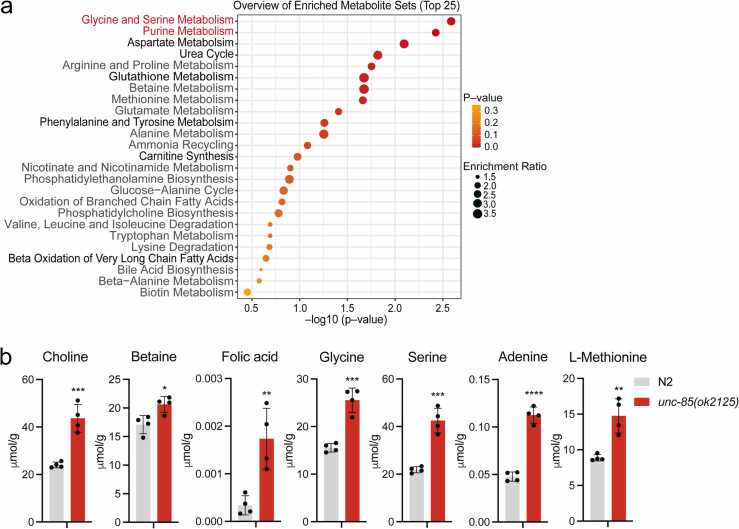
Loss of UNC-85 leads to elevated levels of multiple OCM-associated metabolites. (a) SMPDB-terms enriched among the metabolites showing significantly different levels in *unc-85(ok2125)* mutants compared to N2. The enrichment analysis was performed using MetaboAnalyst 5.0.^41^ (b) Bar graphs comparing the levels of selected OCM-related metabolites in *unc-85(ok2125)* mutants and N2 (*P < 0.05, **P < 0.01, ***P < 0.001, ****P < 0.0001, unpaired t-test). Abbreviations used: OCM, one-carbon metabolism; SMPDB, small molecule pathway database; OCM; One-Carbon Metabolism; SMPDB, Small Molecule Pathway Database.

Cellular metabolites play a crucial role in modulating the epigenetic landscape, influencing processes such as chromatin remodeling and histone modifications.^39^ Conversely, alterations in multiple epigenetic mechanisms are associated with metabolic diseases.^40^ Therefore, considering that loss of UNC-85 presumably affects histone metabolism and thereby chromatin structure/function, we sought to investigate whether UNC-85-depleted animals exhibit altered metabolism, which could, at least partly, explain their shortened lifespan (Figure 1(a)). To explore this, we conducted a metabolomic analysis of N2 and *unc-85(ok2125)* mutants (Supplementary Material File 2). This analysis was performed with L4-stage animals to exclude the oogenesis-derived and embryogenesis-derived metabolomic effects. A subsequent t-test revealed significant differences in 64 metabolites between the 2 strains (Supplementary Material File 1, Table S2). The enrichment analysis with MetaboAnalyst 5.0^41^ revealed that *glycine and serine metabolism* and *purine metabolism* are the two most enriched Small Molecule Pathway Database-terms (Figure 3(a)). These metabolic pathways are strongly associated with one-carbon metabolism (OCM), which supports processes such as purine and thymidine biosynthesis, and epigenetic maintenance.^42^, ^43^ Furthermore, among the 64 metabolites (Supplementary Material File 1, Table S2), many compounds that exhibit elevated levels in *unc-85(ok2125)* mutants serve as OCM substrates, cofactors, or products^42^ (Figure 3(b)). Notably, folate metabolism, the central part of OCM, consists of different intermediates with very similar structures,^43^ but this analysis did not separate them. Therefore, the term “folic acid” (Figure 3(b)) comprises all different folate species. Nevertheless, these data suggest that the increase in OCM activity is one of the major metabolomic changes caused by the loss of UNC-85.

### Knockdown of the OCM component partially rescues the short lifespan of *unc-85(ok2125)* mutants

**Fig. 4:**
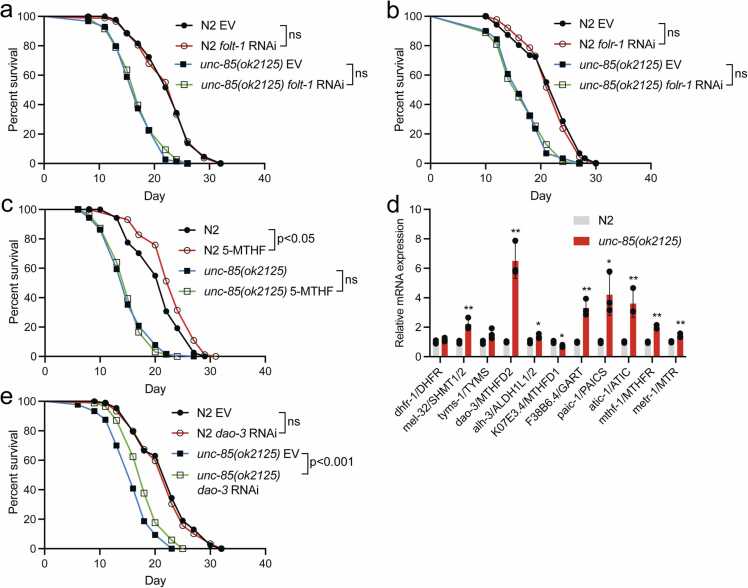
Unlike the knockdown of folate transporters or folate supplementation, RNAi of OCM component *dao-3* partly rescues the lifespan of *unc-85(ok2125)* mutants. (a) Lifespan of N2 and *unc-85(ok2125)* mutants on *folt-1* and (b) *folr-1* RNAi. (c) The effect of 5-MTHF supplementation (100 nM) on the lifespan of N2 and *unc-85(ok2125)* mutants. (d) qRT-PCR of OCM-related genes in L4 stage N2 and *unc-85(ok2125)* mutants. Bars represent mRNA levels relative to N2 with error bars indicating mean ± SD of three biological replicates, each with three technical replicates (*P< 0.05, **P < 0.01, unpaired t-test). (e) Lifespan of N2 and *unc-85(ok2125)* mutants on *dao-3* RNAi. Statistical calculations for lifespan experiments were performed using the Cox-proportional hazard regression analysis. Lifespan statistics are reported in Supplementary Material File 1, Table S1. Abbreviations used: EV, empty vector; 5-MTHF, 5-methyltetrahydrofolate; ns, not significant; OCM, one-carbon metabolism; qRT-PCR, quantitative RT-PCR; RNAi, RNA interference; SD, standard deviation; DAO-3, Methenyltetrahydrofolate cyclohydrolase; FOLR-1, FOLate Receptor homolog; qRT-PCR, quantitative Reverse Transcription Polymerase Chain Reaction; SD, Standard Deviation.

Excessive folate synthesis within the microbiome has been shown to limit *C. elegans* lifespan.^44^, ^45^ Considering the elevated levels of OCM-associated metabolites observed in *unc-85(ok2125)* mutants (Figure 3(a) and (b)), we hypothesized that these animals exhibit elevated folate uptake from the microbiome. Therefore, we investigated how the inhibition of folate uptake affects their lifespan. Specifically, we employed RNAi against Folate transporter 1 (*folt-1*) (a homolog of the reduced folate carrier)^46^ and *folr-1* (homolog of folate receptor).^48^, ^47^ Despite their established contribution to folate uptake in *C. elegans*, neither treatment was found to affect the lifespan of N2 or *unc-85(ok2125)* mutants (Figure 4(a) and (b)). As a side note, we have previously demonstrated that *folt-1* RNAi increases amyloid beta accumulation in a *C. elegans* model of amyloid toxicity.^48^ This, combined with the lifespan data presented here (Figure 4(a)), establishes that FOLT-1 regulates protein homeostasis through a mechanism independent of processes that modulate aging. Contrary to the downregulation of folate transporters, we explored whether increasing folate levels would influence the lifespan of *unc-85(ok2125)* mutants. To test this, we supplemented plates with 100 nM of 5-methyltetrahydrofolate (5-MTHF), an intermediate in OCM previously shown to positively impact lifespan.^48^ Intriguingly, while 5-MTHF extends the lifespan of N2, it has no effect on the lifespan of *unc-85(ok2125)* mutants (Figure 4(c)). This result supports the model that UNC-85-deficient animals exhibit elevated OCM activity and that its further enhancement with supplemented folate has no detectable effect.

Next, we conducted qRT-PCR to analyze whether the loss of UNC-85 affects the mRNA levels of OCM components. Strikingly, *unc-85(ok2125)* mutants exhibited increased expression of many of these genes (Figure 4(d)), with *dao-3*/*MTHFD2*, *F38B6.4*/*GART*, *paic-1*/*PAICS*, and *atic-1*/*ATIC* showing the most pronounced induction. Notably, *dao-3*/*MTHFD2*, a mitochondrial enzyme with bifunctional methylenetetrahydrofolate dehydrogenase/cyclohydrolase activity, is a regulator of purine synthesis,^49^ whereas *F38B6.4*/*GART*, *paic-1*/*PAICS*, and *atic-1*/*ATIC* are downstream components on the purine synthesis pathway.^50^, ^43^ Therefore, the qRT-PCR data support the metabolomic analysis, which revealed the enrichment of purine metabolism in *unc-85(ok2125)* mutants (Figure 3(a)). Given the upregulation of OCM-associated metabolites and gene expression in *unc-85(ok2125)* mutants (Figures 3(a) and 4(d)), we asked whether the downregulation of OCM component affects the lifespan of UNC-85-deficient animals. Our attention was focused on DAO-3/MTHFD2, which has been shown to be the most overexpressed metabolic gene in 19 different tumor types^51^ and whose expression is associated with malignant phenotypes and poor prognosis in different cancers.^52^ The previous study has shown that *dao-3* RNAi administered from the L4 stage does not impact longevity.^53^ We replicated this finding with our RNAi regimen (see Methods), showing that *dao-3* RNAi does not affect the lifespan of N2 animals (Figure 4(e)). However, *dao-3* knockdown significantly extends the lifespan of *unc-85(ok2125)* mutants (Figure 4(e)). Taken together, the data presented here suggest that UNC-85-deficient animals have an overactive OCM, which contributes to their shortened lifespan.

## Discussion

We demonstrate here that both *C. elegans* ASF1 homologs, ASFL-1 and UNC-85, are essential for normal lifespan (Figure 1(a)). Notably, the knockdown of *unc-85* in an *asfl-1* mutant background results in embryonic lethality,^23^ highlighting the compensatory functions of these two factors. The compensatory nature of the two ASF1 homologs provides a unique system for studying how reduced ASF1 activity, using either *asfl-1* or *unc-85* mutants, modulates the organism's physiology. As mentioned earlier, we focused on UNC-85 because it has been shown to possess many conserved ASF1-related features: it is a target of TLK-1 kinase,^30^ it is associated with cell replication,^22^, ^24^, ^25^ and it regulates histone H3–H4 levels in the chromatin.^35^ Given that *cdl-1* RNAi-mediated reduction in histone synthesis does not shorten the lifespan of *unc-85(ok2125)* mutants (Figure 1(c)), and the loss of UNC-85 does not lead to a reduction in total histone levels (Figure S2), it is likely that the shortened lifespan observed in *unc-85(ok2125)* mutants is attributed to reduced nucleosomal histone deposition. Regarding the effect of adult-only *cdl-1* RNAi on lifespan (Figure 1(c)), an important conclusion can be drawn from this experiment: although all somatic cells are post-mitotic in adult *C. elegans*, histone synthesis is still required for normal lifespan. This supports the widely accepted view that nucleosomal histone loss is an important driver of aging.^4^, ^3^, ^5^, ^6^, ^7^

The discovery that UNC-85 is essential for the extended lifespan upon *ash-2* RNAi (Figure 2(a)) is intriguing as it leads to the hypothesis that this histone chaperone is a key regulator of epigenome-mediated longevity. Notably, a previous study has shown that moderate chromatin stress promotes longevity in *C. elegans.*^54^ Therefore, it is possible that the subtle knockdown of H3K4 methyltransferase triggers a lifespan-extending chromatin stress, which, in turn, enhances UNC-85 activity by promoting its nuclear translocation (Figures 2(b) and (c)). In support of UNC-85 being a factor responding to chromatin stress, ASF1 has been shown to be essential for the chromatin-related stress response by buffering excess histone levels and enhancing chromatin assembly in cells exposed to replication stress.^31^ It is tempting to hypothesize that *ash-2* RNAi-mediated alterations in the H3K4me3 pattern cause an imbalance in histone H3 modifications, which is resolved by the increased UNC-85-mediated nucleosomal deposition of newly synthesized histones. Consequently, enhanced nucleosomal histone exchange promotes longevity through epigenetic rejuvenation, which would open new possibilities in anti-aging medicine.

As OCM provides one-carbon units for nucleotide biosynthesis,^42^, ^43^ the increased expression of OCM genes associated with the purine synthesis (Figure 4(d)) and upregulation of glycine, serine, and purine metabolism in *unc-85(ok2125)* mutants (Figure 3(a)) suggest that these animals have an activated response mechanism, which attempts to enhance DNA synthesis to counteract the deficiency in nucleosomal histone deposition. This condition mirrors the metabolism observed in cancer cells, which have a large demand for serine-derived one-carbon units to maintain sufficient nucleotide biosynthesis required for growth and proliferation.^55^ However, in contrast to cancer cells, an excessively active OCM further shortens the short lifespan of *unc-85(ok2125)* mutants, which is evidenced by the finding that *dao-3* RNAi extends the lifespan of UNC-85-deficient animals without affecting N2 animals (Figure 4(e)). As mentioned earlier, all somatic cells in adult *C. elegans* are post-mitotic. Therefore, it is plausible that while an overactivated OCM proves advantageous for rapidly proliferating cells, it may be detrimental for the maintenance of non-dividing cells. This hypothesis gains support from the discovery that long-lived *C. elegans* mutants exhibit reduced levels of many OCM metabolites.^53^

In summary, based on our data, we have made three hypotheses explaining the observed UNC-85-associated phenotypes. Firstly, it is hypothesized that UNC-85-deficient animals experience a shortened lifespan due to impaired nucleosomal histone deposition. Secondly, it is proposed that OCM is upregulated in UNC-85-deficient animals as a compensatory mechanism to counteract histone loss-induced chromatin defects by enhancing nucleotide synthesis. Thirdly, it is suggested that epigenome-targeted interventions promoting longevity, such as the downregulation of H3K4 methyltransferase ASH-2, trigger a chromatin stress response that rejuvenates chromatin, partly through promoting ASF1/UNC-85-mediated supply of newly synthesized histones. As the concept of epigenetic rejuvenation gains traction in aging research, the data presented here should encourage further investigation into the roles of ASF1 and other histone chaperones in this process.

## Methods

### *C. elegans* strains and maintenance

For all experiments, *C. elegans* were maintained on nematode growth medium (NGM) plates (peptone, P4963, Merck; agar, A4550, Merck; NaCl, 746398, Merck) seeded with *Escherichia coli* (*E. coli*) HT115 bacteria carrying the EV (control vector for RNAi). The N2 (Bristol) strain served as the wild-type. N2, *unc-85(ok2125)* II (VC1690), and *asfl-1(ok2060)* I (RB1662) strains were obtained from the Caenorhabditis Genetics Center. The *unc-85(ok2125)* II (VC1690) and *asfl-1(ok2060)* I (RB1662) strains were outcrossed five times with N2. *unc-85(syb3386)*[*unc-85:wrmScarlet*] II (PHX3386) was created using clustered regularly interspaced short palindromic repeats/CRISPR associated protein 9 (CRISPR/Cas9)-mediated genome editing (SunyBiotech). The intestine-specific *unc-85* OE strains (PHX8481 *sybIs8481[Pges-1:unc-85:unc-85 3′UTR, Pmyo-2:gfp]* and PHX8486 *sybIs8486[Pges-1:unc-85:unc-85 3′UTR, Pmyo-2:gfp]*) were created using microinjection (SunyBiotech). Transgenes were integrated into the genome through gamma irradiation (SunyBiotech). PHX8481 and PHX8486 strains were outcrossed five and four times with N2, respectively. The sequences related to PHX3386, PHX8481, and PHX8486 can be found in Supplementary Material File 1.

### RNA interference

Cloning of the *folt-1* RNAi has been described earlier.^48^ *cdl-1*, *tlk-1*, *ash-2 folr-1, dao-3*, and *cox-5B* RNAi clones were taken from the Ahringer RNAi library. RNAi was performed using the feeding protocol described earlier.^56^

### Hydroxyurea treatment

Hydroxyurea was added to NMG media at a final concentration of 25 mM. At the L4 stage, *unc-85(syb3386)*[*unc-85:wrmScarlet*] II (PHX3386) animals were transferred to HU-containing plates and imaged 24 h later using an Olympus BX63 microscope.

### Imaging

*unc-85(syb3386)*[*unc-85:wrmScarlet*] II (PHX3386) animals were mounted on 2% agarose pads and immobilized using levamisole hydrochloride (30 μM) diluted in M9 solution. Imaging was done with an Olympus BX63 microscope using a 20× objective. The intestinal nuclei containing a distinguishable signal from UNC-85:wrmScarlet were counted from individual images. The UNC-85:wrmScarlet signal in HU-treated animals was quantified using ImageJ. For each imaged animal, the UNC-85:wrmScarlet signal was quantified from one gonad arm (the one in good focus) and a representative area of the embryos inside the animal.

### Lifespan analyses

All lifespan experiments were performed at 20 °C on *E. coli* HT115 containing either the EV or RNAi vector. Lifespan experiments were initiated by letting gravid hermaphrodites (P0 generation) to lay eggs on NGM agar plates, and the F1 generation was scored for lifespan. Alternatively, animals were bleached and let to hatch overnight in M9 before plating L1 larvae to experimental plates. These two alternative ways to initiate lifespan did not affect the conclusions made from the experiments. At the L4 larval stage animals were transferred to plates containing 5-Fluorouracil (10 µM) (Merck, #F6627) to prevent progeny production. *cdl-1* RNAi was initiated on day 1 of adulthood (animals kept on EV during development). For *ash-2*, *folt-1*, *folr-1*, and *dao-3* RNAi lifespan experiments, animals of P0 generation were placed on RNAi plates as L3 larvae and their progeny (F1 generation) was used for lifespan assays. This RNAi regimen was used to improve the knockdown efficiency. For 5-MTHF (Merck, #M0132) supplementation experiments, 5-MTHF was added to NMG media at a final concentration of 100 nM. Animals that had an exploded vulva or that crawled off the plate were censored. Animals were counted as dead if there was no movement after poking with a platinum wire. Lifespans were checked every 1–3 days. Mean lifespan ± standard error is reported in the Supplementary Material File 1, Table S1.

### Targeted semiquantitative 100 metabolite analysis

N2 and *unc-85(ok2125)* animals were synchronized by bleaching and plated as L1 larvae on NGM agar plates seeded with *E. coli* HT115 (EV). Plates were kept at 20 °C. Animals were harvested at the L4 stage (approximately 6000 animals/sample) and frozen in liquid nitrogen. Metabolites were extracted from frozen samples in Precellys homogenization tubes (Precellys 24 lysing kit, Precellys) containing 1.4 mm ceramic (Zirconium oxide) beads. At this point, 16 μL of labeled internal standard (IS) mix (Cambridge Isotope Laboratory) was added, followed by a 10-min incubation on ice. Next, two homogenization steps were performed. In the first step, 400 µL of precooled 99% acetonitrile (ACN) + 1% formic acid (FA) was added, followed by centrifugations (3 cycles of 20 s, 5500 rpm, with a 10 s break in between, and after these, 10 min at 14,000 rpm at 4 °C). The supernatant was collected in a 1.5 mL Eppendorf tube. In the second step, 400 µL of extraction solvent (90:10, ACN:Milli-Q H_2_O + 1% FA, HiPerSolv CHROMANORM, HPLC grade, VWR Chemicals BDH) was added to the remaining pellet, and the steps described above were repeated. The extracts from the two homogenization steps were pooled. The collected supernatant was dispensed into an OstroTM 96-well plate (Waters Corporation, Milford, USA) and filtered on a Hamilton robot's vacuum station. Finally, 5 µL of filtered sample extract was injected into the Waters liquid chromatography-mass spectrometry system, and 100 metabolites were separated using Waters Acquity ultra-performance liquid chromatography and analyzed using triple quadrupole mass spectrometry, as previously described.^57^, ^58^ The column oven temperature and autosampler temperature were set to 40 °C ± 3 °C and 5 °C ± 3 °C, respectively. The multiple reaction monitoring acquisition mode was selected for the quantification of metabolites. The calibration solutions were prepared in a 96-well plate by serial dilution of the stock calibration mix using Hamilton’s MICROLAB STAR line (Hamilton, Bonaduz AG, Switzerland) liquid handling robot system. MassLynx 4.1 software was used for data acquisition, data handling, and instrument control. Data processing was done using TargetLynx software. The data are presented as concentration (µmol\g) (see Figure 3(b) and Supplementary Material File 2).

### Quantitative RT-PCR

Animals were synchronized by bleaching and plated as L1 larvae on NGM agar plates seeded with *E. coli* HT115 (EV). Plates were kept at 20 °C. Animals were collected at the L4 stage and frozen in liquid nitrogen. TRIzol Reagent (Thermo Fisher Scientific, #15596018) was used to extract RNA. cDNA (complementary DNA) synthesis was done with the QuantiTect Reverse Transcription Kit (Qiagen, #205313) and quantitative reverse transcription polymerase chain reaction (qRT-PCR) reactions were run with the HOT FIREPol SolisGreen qPCR Mix-reagent (Solis BioDyne, #08-46-00001) using the CFX384 machine (Bio-Rad). qRT-PCR data were normalized to the expression of *cdc-42* and *pmp-3*. qRT-PCR oligos used in this study are provided in Supplementary Material File 1, Table S3. qRT-PCR experiments were performed with three biological replicates, with three technical replicates for each biological replicate. Statistical significances were analyzed using an unpaired t-test.

### Western blot

Animals were synchronized by bleaching and plated as L1 larvae on NGM agar plates seeded with *E. coli* HT115 (EV). Plates were kept at 20 °C. Animals were collected at the L4 stage and frozen in liquid nitrogen. Animals were lysed in a protease inhibitor cocktail (Thermo Fisher Scientific, #78430)-supplemented urea solution (Merck, #51457) by grinding with a plastic pestle in 1.5 mL Eppendorf tubes. Lysates were resolved on 4–15% precast polyacrylamide gels (Bio-Rad, #4561083). Immun-Blot polyvinylidene fluoride (PVDF) Membrane (Bio-Rad, #1620177) was used for blotting. The histone H3 antibody (PA5-16183), Histone H2A antibody (PA5-35893), Histone H2B antibody (PA1-41058), Histone H4 antibody (PA5-85583), and H3K4me3 antibody (MA5-11199) were purchased from Thermo Fisher Scientific (used with a 1:1000 dilution). The α-tubulin antibody (used with a 1:5000 dilution) was purchased from Merck (#T5168). Clarity Western enhanced chemiluminescence (ECL) Substrate (Bio-Rad, #1705061) and ChemiDoc MP-imager (Bio-Rad) were used for protein detection. Western blot signal intensities were quantified using ImageJ.

### Statistical analysis

Statistical analyses for qRT-PCR data were carried out in GraphPad Prism and Excel, and the data represent the mean of three biological replicates ± standard deviation. Statistical details can be found in the figures and figure legends. Statistical calculations for lifespan experiments were carried out in R using the Cox-proportional hazard regression analysis. Statistical details for the lifespan data can be found in Supplementary Material File 1, Table S1.

## Declarations of interest

The authors declare that they have no known competing financial interests or personal relationships that could have appeared to influence the work reported in this paper.

## Data Availability

Data will be made available on request.
