# Supplementary material for: Loss of the histone chaperone UNC-85/ASF1 inhibits the epigenome-mediated longevity and modulates the activity of one-carbon metabolism
Source: Cell Stress Chaperones. 2024 Apr 10;29(3):392–403. doi: 10.1016/j.cstres.2024.04.003 (PMC11039323; doi:10.1016/j.cstres.2024.04.003)
Supplement: Supplementary file 1 — Supplementary material [file mmc1.pdf]

Cell Stress and Chaperones

Loss of the histone chaperone UNC-85/ASF1 inhibits the epigenome-mediated longevity and modulates the activity of one-carbon metabolism

Bideep Shrestha (ORCID: 0000-0002-0122-0705)[1], Anni I. Nieminen (ORCID: 0000-0001-8999-6040)[2], Olli Matilainen (ORCID: 0000-0001-9939-4828)[1]*

[1]The Molecular and Integrative Biosciences Research Programme, Faculty of Biological and Environmental Sciences, University of Helsinki, Finland

[2]FIMM Metabolomics Unit, Institute for Molecular Medicine Finland, University of Helsinki, Finland

* Correspondence: olli.matilainen@helsinki.fi

Supplementary Material file 1

- **Figures S1 and S2**
- **Tables S1, S2, and S3**
- **Sequence related to the strains PHX3386, PHX8481 and PHX8486**

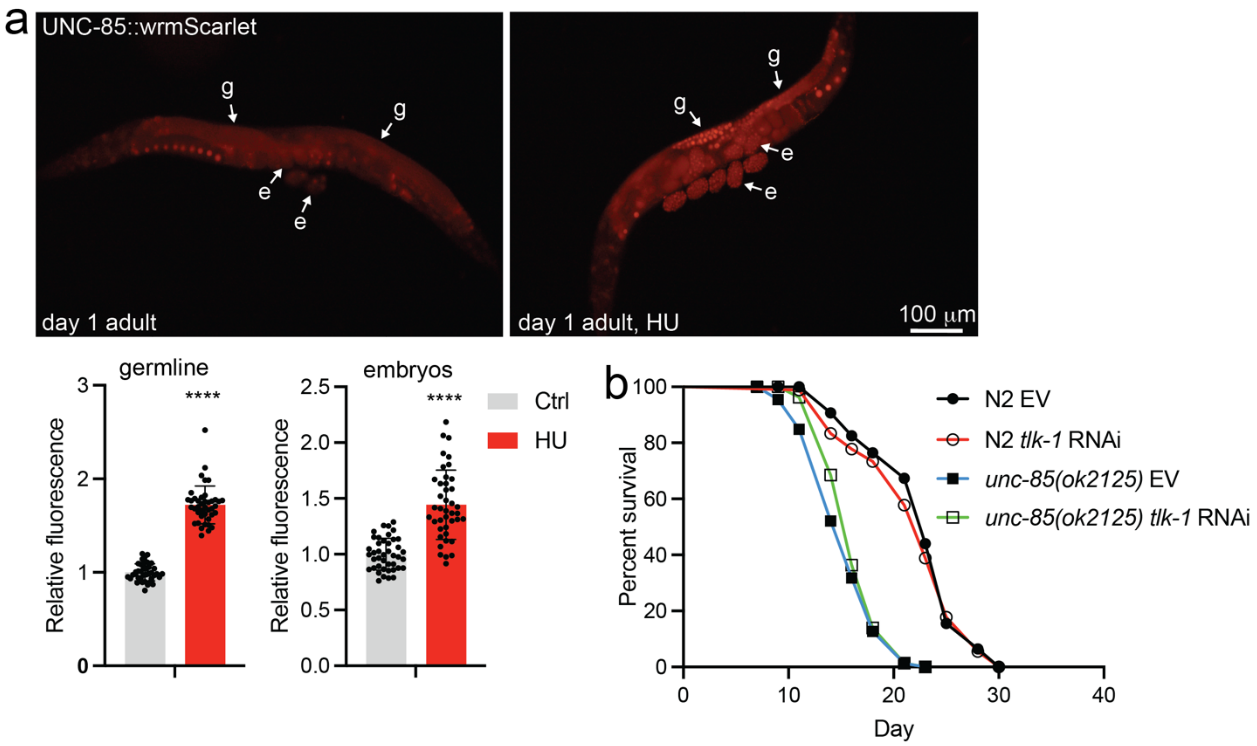

Fig. S1 (**a**) Images of untreated and hydroxyurea (HU)-treated day 1 adult UNC-85::wrmScarlet-expressing animals (PHX3386). Arrows indicate tissues that show HU-induced accumulation of the UNC-85::wrmScarlet-fusion protein. Abbreviations: g, germline; e, embryo. Bar graph shows the quantification of UNC-85::wrmScarlet signal in germline and embryos (n = 42 animals for Ctrl and n = 41 animals for HU-treated animals, data collected from two independent experiments, ****$p < 0.0001$, unpaired t-test). (**b**) Lifespan of N2 and *unc-85(ok2125)* mutants on *tlk-1* RNAi. Statistical calculations for lifespan experiments were performed using the Cox-proportional hazard regression analysis. Lifespan statistics are reported in Supplementary Material file 1, Table S1

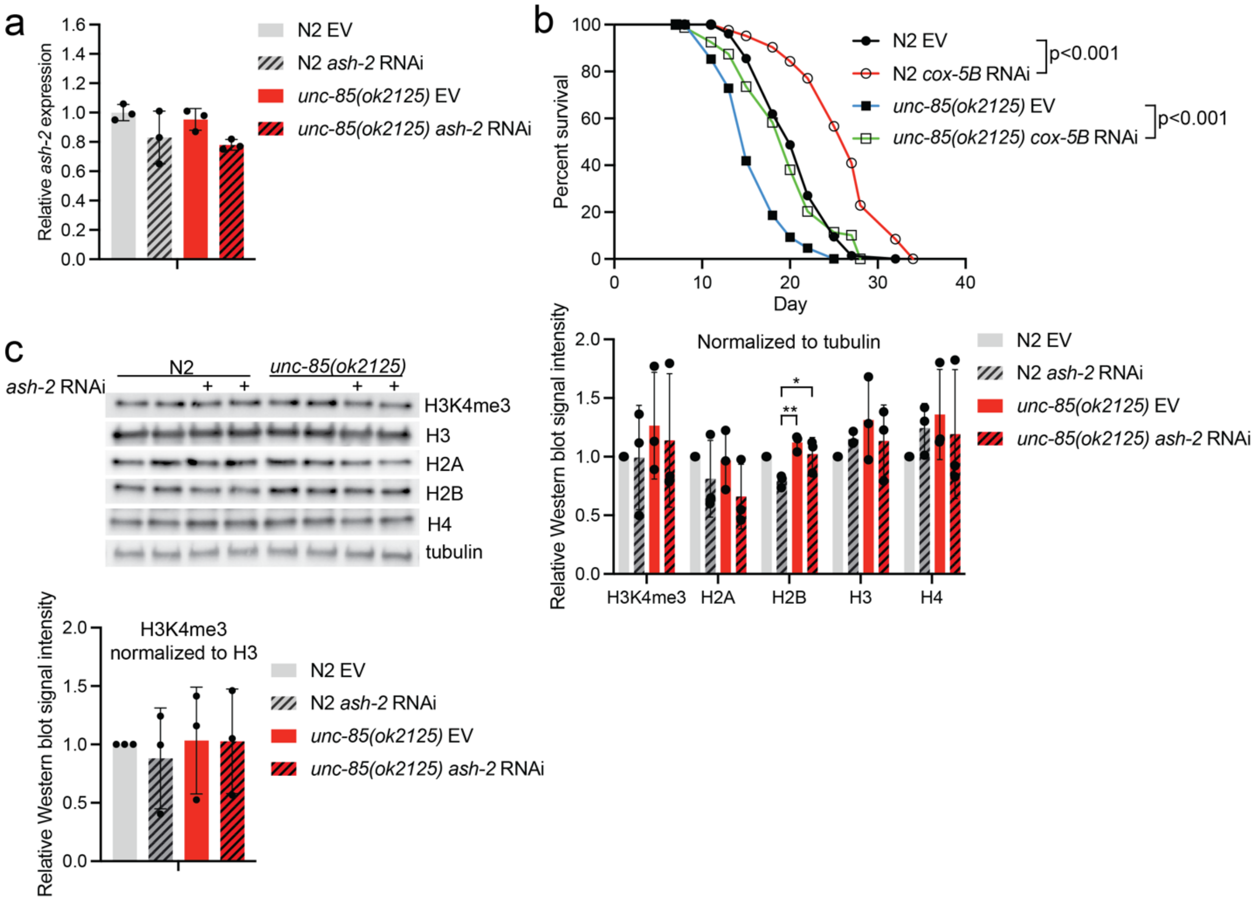

Fig. S2 (**a**) qRT-PCR analysis of *ash-2* mRNA in L4 larval stage N2 and *unc-85(ok2125)* mutants upon *ash-2* RNAi (RNAi treatment initiated at L1 larval stage). Bars represent mRNA levels relative to EV (empty vector)-treated N2 with error bars indicating mean ± SD of three biological replicates, each with three technical replicates. In this experiment, differences are not significant based on one-way ANOVA. Using a t-test to compare only N2 EV vs. N2 *ash-2* RNAi and *unc-85(ok2125)* EV vs. *unc-85(ok2125) ash-2* RNAi, the difference between *unc-85(ok2125)* EV vs. *unc-85(ok2125) ash-2* RNAi is statistically significant ($p < 0.05$). (**b**) Lifespan of N2 and *unc-85(ok2125)* mutants on *cox-5B* RNAi. Statistical calculations for lifespan experiments were performed using the Cox-proportional hazard regression analysis. Lifespan statistics are reported in Supplementary Material file 1, Table S1. (**c**) Western blot of H3K4me3 and core histones from

the lysates of L4 stage N2 and *unc-85(ok2125)* mutants. Bar graphs shows quantifications from three independent experiments (*p < 0.05, **p < 0.01, one-way ANOVA with Tukey's test)

Table S1. Individual replicates of *C. elegans* lifespan experiments

Genotype, RNAi and treatment	mean lifespan ± SE (days)	variation compared to control (%)	*P*-values against control	N
**Figure 1a**				
N2 (HT115, EV)	21.8 ± 0.67			83
asfl-1(ok2060) (HT115, EV)	19.5 ± 0.51	- 10.6	4.54e-05	82
unc-85(ok2125) (HT115, EV)	19.3 ± 0.54	- 11.5<br>& - 1	1.19e-05<br>& 0.534	68
N2 (HT115, EV)	21.5 ± 0.63			84
asfl-1(ok2060) (HT115, EV)	17.2 ± 0.41	- 20	3.72e-10	83
unc-85(ok2125) (HT115, EV)	17 ± 0.5	- 20.9<br>& - 1.2	1.47e-09<br>& 0.674	80
**Figure 1c**				
N2 EV	22.8 ± 0.53			85
N2 *cdl-1* RNAi	19.7 ± 0.41	- 13.6	4.87e-07	85
unc-85(ok2125) EV	17.8 ± 0.47	- 21.9	3.80e-12	88
unc-85(ok2125) cdl-1 RNAi	17.5 ± 0.41	- 23.2<br># - 1.7	7.73e-15<br># 0.3	84

N2 EV	22 ± 0.47			81
N2 *cdl-1* RNAi	19.7 ± 0.35	- 10.5	3.40e-05	79
unc-85(ok2125) EV	15.8 ± 0.55	- 28.2	3.66e-16	50
unc-85(ok2125) cdl-1 RNAi	16.2 ± 0.38	- 26.4<br># + 2.5	< 2e-16<br># 0.634	58
**Figure 2a**				
N2 EV	22.3 ± 0.63			79
N2 *ash-2* RNAi	24.6 ± 0.65	+ 9.3	0.000327	80
unc-85(ok2125) EV	17.9 ± 0.62	- 19.7	1.51e-06	66
unc-85(ok2125) ash-2 RNAi	17 ± 0.49	- 23.8<br># - 5	2.79e-11<br># 0.134	80
N2 EV	20.8 ± 0.45			88
N2 *ash-2* RNAi	23.4 ± 0.42	+ 11.1	0.00012	87
unc-85(ok2125) EV	16.6 ± 0.34	- 20.2	2.97e-16	90
unc-85(ok2125) ash-2 RNAi	16.3 ± 0.38	- 21.6<br># - 1.8	< 2e-16<br># 0.742	90
**Figure 2d**				
N2 EV	21 ± 0.5			95
unc-85 OE (PHX8481)	22.8 ± 0.35	+ 7.9	0.338	93
unc-85 OE (PHX8486)	22.7 ± 0.37	+ 7.5	0.159	94
N2 EV	21.2 ± 0.54			88
unc-85 OE (PHX8481)	22.6 ± 0.37	+ 6.2	0.884	85
unc-85 OE (PHX8486)	22.5 ± 0.45	+ 5.8	0.412	85

**Figure 4a**				
N2 EV	22.8 ± 0.5			87
N2 *folt-1* RNAi	22.6 ± 0.52	- 0.9	0.957	86
unc-85(ok2125) EV	17.0 ± 0.43	- 25.4	< 2e-16	76
unc-85(ok2125) folt-1 RNAi	17.4 ± 0.44	- 23.7<br># + 2.3	9.07e-15<br># 0.	77
N2 EV	18.4 ± 0.47			79
N2 *folt-1* RNAi	19.7 ± 0.44	+ 6.6	0.168	83
unc-85(ok2125) EV	14.1 ± 0.31	- 23.4	1.06e-14	88
unc-85(ok2125) folt-1 RNAi	13.6 ± 0.32	- 26.1<br># - 3.5	< 2e-16<br># 0.425	88
**Figure 4b**				
N2 EV	21.7 ± 0.51			88
N2 *folr-1* RNAi	22.5 ± 0.47	+ 3.6	0.428	87
unc-85(ok2125) EV	16.5 ± 0.45	- 24	6.32e-13	90
unc-85(ok2125) folr-1 RNAi	16.6 ± 0.46	- 23.5<br># + 0.6	1.41e-12<br># 0.944	89
N2 EV	22.1 ± 0.53			87
N2 *folr-1* RNAi	22.1 ± 0.45	0	0.549	89
unc-85(ok2125) EV	16.8 ± 0.43	- 24	1.54e-14	89
unc-85(ok2125) folr-1 RNAi	16.7 ± 0.46	- 24.4<br># - 0.6	2.87e-14<br># 0.947	87
**Figure 4c**				

N2	20.9 ± 0.49			80
N2 5-MTHF (100 nM)	23.1 ± 0.44	+ 9.5	0.0041	85
unc-85(ok2125)	15.2 ± 0.42	- 27.3	< 2e-16	82
unc-85(ok2125) 5-MTHF (100 nM)	15.2 ± 0.36	- 27.3<br>@ 0	< 2e-16<br>@ 0.753	80
N2	20 ± 0.5			83
N2 5-MTHF (100 nM)	21.5 ± 0.57	+ 7	0.0326	78
unc-85(ok2125)	16.9 ± 0.38	- 15.5	1.02e-08	82
unc-85(ok2125) 5-MTHF (100 nM)	17.1 ± 0.33	- 14.5<br>@ + 1.2	6.80e-09<br>@ 0.965	81
N2	20.5 ± 0.55			63
N2 5-MTHF (100 nM)	21.9 ± 0.59	+ 6.4	0.0343	70
unc-85(ok2125)	15.6 ± 0.5	- 23.9	8.16e-11	49
unc-85(ok2125) 5-MTHF (100 nM)	15.8 ± 0.51	- 22.9<br>@ + 1.3	1.59e-09<br>@ 0.879	42

Figure 4e

N2 EV	22.2 ± 0.53			85
N2 *dao-3* RNAi	21.8 ± 0.52	- 1.8	0.691	89
unc-85(ok2125) EV	16.1 ± 0.41	- 27.5	< 2e-16	86
unc-85(ok2125) dao-3 RNAi	18.2 ± 0.38	- 18<br># + 11.5	5.02e-10<br># 0.000992	85
N2 EV	21.7 ± 0.52			88
N2 *dao-3* RNAi	21.8 ± 0.52	+ 0.5	1	88

unc-85(ok2125) EV	16.6 ± 0.4	- 23.5	< 2e-16	89
unc-85(ok2125) dao-3 RNAi	18.8 ± 0.38	- 13.4<br># + 11.7	8.21e-08<br># 0.000382	82
**Figure S1b**				
N2 EV	22.6 ± 0.49			79
N2 *tlk-1* RNAi	22.0 ± 0.52	- 2.7	0.654	90
unc-85(ok2125) EV	15.5 ± 0.35	- 31.4	< 2e-16	81
unc-85(ok2125) tlk-1 RNAi	16.4 ± 0.3	- 27.4<br># + 5.5	< 2e-16<br># 0.281	73
N2 EV	20.0 ± 0.5			83
N2 *tlk-1* RNAi	20.3 ± 0.51	+ 1.5	0.829094	78
unc-85(ok2125) EV	16.9 ± 0.38	- 15.5	3.01e-08	82
unc-85(ok2125) tlk-1 RNAi	18.1 ± 0.37	- 9.5<br># + 6.6	0.000166<br># 0.0505	80
**Figure S2b**				
N2 EV	20.8 ± 0.46			75
N2 *cox-5B* RNAi	26.2 ± 0.55	+ 20.6	3.43e-12	83
unc-85(ok2125) EV	16.1 ± 0.44	- 22.6	4.51e-10	65
unc-85(ok2125) cox-5B RNAi	19.6 ± 0.54	- 5.8<br># + 17.9	0.797<br># 7.13e-07	79

& Compared to *asfl-1(ok2060)* (HT115, EV)

# Compared to *unc-85(ok2125)* EV

@ Compared to untreated *unc-85(ok2125)*

Table S2. 64 compounds whose levels were found to differ between N2 and *unc-85(ok2125)* mutants in metabolomic analysis (t-test, p < 0.05)

Sr No.	Name of compound	t-stat	p-value	-LOG10(p)	FDR
1	Phosphoethanolamine	-16.065	3.70E-06	5.4322	0.000336
2	Adenine	-13.454	1.04E-05	4.981	0.000475
3	Isovalerylcarnitine	-11.319	2.85E-05	4.5458	0.000863
4	Serine	-9.7499	6.69E-05	4.1745	0.001006
5	Arginine	-9.2903	8.80E-05	4.0555	0.001006
6	Guanidinoacetic Acid	-9.2608	8.96E-05	4.0477	0.001006
7	Tryptophan	-9.1604	9.53E-05	4.021	0.001006
8	L-Glutamic Acid	-9.1148	9.80E-05	4.0088	0.001006
9	Isobutyrylcarnitine	-9.0906	9.95E-05	4.0022	0.001006
10	Propionylcarnitine	-8.8432	0.00011622	3.9347	0.001058
11	Choline	-8.366	0.00015861	3.7997	0.001254
12	Glycine	-8.2733	0.00016879	3.7727	0.001254
13	Niacinamide	-8.1362	0.00018525	3.7322	0.001254
14	Tyrosine	-8.0043	0.00020286	3.6928	0.001254
15	Octanoylcarnitine	-7.9278	0.00021396	3.6697	0.001254
16	Acetylcarnitine	-7.8848	0.0002205	3.6566	0.001254
17	Leucine	-7.6514	0.00026031	3.5845	0.001393
18	Threonine	-7.3284	0.00032988	3.4816	0.00156
19	Phenylalanine	-7.3065	0.00033533	3.4745	0.00156
20	Pantothenic Acid	-7.2766	0.00034291	3.4648	0.00156
21	Asparagine	-7.1521	0.00037679	3.4239	0.001633
22	Glutamine	-6.6494	0.00055888	3.2527	0.002312
23	IMP	6.5062	0.00062799	3.202	0.002485
24	Cytosine	-6.1354	0.00085769	3.0667	0.003252
25	Chenodeoxycholic Acid	-5.9709	0.00098946	3.0046	0.003602
26	Cytidine	-5.9076	0.0010462	2.9804	0.003662
27	L-Methionine	-5.7853	0.0011667	2.933	0.003823
28	Proline	-5.7762	0.0011763	2.9295	0.003823
29	Citrulline	5.469	0.0015594	2.807	0.004893
30	Valine	-5.4191	0.0016342	2.7867	0.004957
31	Homoserine	-5.3507	0.0017435	2.7586	0.005118
32	L-Kynurenine	-5.2585	0.0019041	2.7203	0.005415
33	Asymmetric dimethylarginine	-5.2201	0.001976	2.7042	0.005449
34	Glyceraldehyde	-5.1079	0.0022042	2.6567	0.005742
35	Nicotinic Acid	-5.106	0.0022083	2.6559	0.005742
36	Kynurenic Acid	-4.9761	0.0025114	2.6001	0.00618
37	deoxycytidine	-4.9756	0.0025127	2.5999	0.00618

38	Folic acid	-4.8969	0.0027193	2.5655	0.006512
39	Adenosine	-4.8248	0.0029257	2.5338	0.006827
40	cAMP	-4.6898	0.0033617	2.4734	0.007648
41	Taurine	-4.1611	0.0059373	2.2264	0.01294
42	Taurochenodeoxycholic Acid	-4.1558	0.0059722	2.2239	0.01294
43	Allantoin	-4.133	0.0061266	2.2128	0.012966
44	Alanine	-4.1041	0.006328	2.1987	0.013088
45	Uracil	-4.0025	0.0070989	2.1488	0.014356
46	2-Aminoisobutyric acid	-3.9751	0.0073243	2.1352	0.014489
47	Hypoxanthine	-3.8436	0.0085233	2.0694	0.016503
48	Neopterin	-3.7924	0.0090483	2.0434	0.017154
49	Isoleucine	-3.7696	0.0092928	2.0319	0.017258
50	GABA	-3.7083	0.0099893	2.0005	0.018181
51	Guanosine	-3.649	0.010719	1.9698	0.019127
52	Aminodipic Acid	-3.4523	0.013595	1.8666	0.023792
53	Inosine	-3.3269	0.015868	1.7995	0.026712
54	Creatine	3.3192	0.01602	1.7954	0.026712
55	Glutathione	-3.313	0.016145	1.792	0.026712
56	Hydroxyproline	-3.2783	0.016858	1.7732	0.027394
57	Betaine	-3.2036	0.018516	1.7325	0.029561
58	Taurocholic Acid	-3.0049	0.023856	1.6224	0.037031
59	Lysine	-3	0.024009	1.6196	0.037031
60	Acetoacetic acid	-2.962	0.025218	1.5983	0.038248
61	Carnitine	-2.9271	0.026386	1.5786	0.039363
62	Decanoylcarnitine	-2.7693	0.032452	1.4888	0.046953
63	Creatinine	-2.7681	0.032506	1.488	0.046953
64	4-Pyridoxic Acid	-2.727	0.034323	1.4644	0.048804

Table S3. Oligonucleotide sequences used in qRT-PCR

Gene	Forward (5’ → 3’)	Reverse (5’ → 3’)
cdc-42	CTGCTGGACAGGAAGATTACG	CTCGGACATTCTCGAATGAAG
pmp-3	GTTCCCGTGTTCATCACTCAT	ACACCGTCGAGAAGCTGTAGA
dgfr-1	GCCGATGTTCATCTCAAGTCA	TCGACAACCTTCCATTTGCA
mel-32	AGGTGGACCACACAATCACA	ATTCTCTCGGCCAAGGTTTT
tyms-1	GAGCAACCGGATAGTCGAAG	CGTTGGTAAAGCTGACACGA
dao-3	TGGTGATGCCAACTTTGTGT	TGCTCTCAGGCATTTCTCCT
alh-3	CTGATGTCGATCGTGCTGTT	TGCTCCAGCATCTAGGGACT
K07E3.4	TTGCTGACAAGGTTGCTTTG	GACAACTGCTGATGGCTGAA
F38B6.4	TGGCGCGAAAAATTTTAACT	GCGCATCCTTTTACAAGACC
paic-1	CATTCAACGCTGTCCGTAAA	ATCGGAATCATGGTGCATTT
atic-1	AGCCAGCAAACAGGAGTTGT	CATCACGAACTGAGCCTCCT
mthf-1	GGGAATCATGCCAATTATGG	TTCAATGCAACGCTCTGTTC
metr-1	GGAAAAATGCTGCTCTCTGG	ACTGTCGACTTGCACACGAG
ash-2	CCCTTATGAGCTCCCATTTTCC	CGAGTTGGCGGGAAATTCAA

Strain name and genotype: PHX3386 *unc-85(syb3386)*[*unc-85::wrmScarlet*] II

Synonymous mutation is labeled in blue. *unc-85* exons are labeled with yellow and orange and wrmScarlet is labeled in red.

>syb3386
ATGGCTTCCCGTGTCAACATTGTTCAAGTACAAATTCTCGACAATCCTGCGATGTTTG
TCGACAAATTCAAGTTGGAGATCACATTTGAAGTTTTTGAGCACTTGCCGCATGgtaag
aaagaggaaaatataaactcatcgataataatgataattttcagATCTCGAATGGGAATTGGTCTACGTCGGATC
CGGAACATCCCGAGACTTTGACCAAGTTCTCGATTCAGCGCTCGTTGGTCCAATTCC
AGAAGGACGCCACAAGTTTGTGTTCGACGCGGATCATCCGGATATCTCGAAGATCCC
AGTCGATGATATCGTCGGTGTCAGCGTACTTCTCCTGCGCTGCAAGTACAACGATCA
GGAGTTTATCAATATGGGATGGTTCGTGGCAAATGAGTACACCGAGGAAGAGCTCA
AAGAGAATCCCCCATCGCAGCCACTCATCGAAAAGCTTTCCCGCAAAGTCGAAACT
GAAGATCTTCGCATCACCACATTCCCGATTCGCTGGACTGATGAGGATCCGGTCGCA
GAGCCTGTCGAGGATGAGGCTAACAGAGTATTCGCGGAAGATGACCTCATGCCTCT
GAATGATGATGGCCAGGAAGATGACGATGAGGAGGAAGAGgtgagaaaatgaattttacttattcaa
aaattaaataattttattcagGATGATGACGAGATGGAGGCCAACGCTGAAGAAGTGGATTTGAA
CGAGAGCTTCAATGAACGATTGGCCAATGCACTTGACGGGGCCGAACAGAAAGGCG
CAGATGAGAAAATGGAGGACGATGGAGCAAATGAGGATGTTGATATGGCTGgtaagtgtt
acttggtttctctaacccccttcttgtttcgtgatgtgtgcttctcaccctgtaattatagATGACGAGCCTGGCGTTCAGATC
AACACTGACACGAAAGTTCCGGAAAGTATGGCGGAGCCATTGTCAGACAAGACAA
ACAACGAAATGGTCCAGATGGTCAGCAAGGGAGAGGCAGTTATCAAGGAGTTCATG
CGTTTCAAGGTCCACATGGAGGGATCCATGAACGGACACGAGTTCGAGATCGAGGG
AGAGGGAGAGGGACGTCCATACGAGGGAACCCAAACCGCCAAGCTCAAGGTCACC
AAGGGAGGACCACTCCCATTCTCCTGGGACATCCTCTCCCCACAATTCATGTACGGA
TCCCGTGCCTTCACCAAGCACCCAGCCGACATCCCAGACTACTACAAGCAATCCTTC
CCAGAGGGATTCAAGTGGGAGCGTGTCATGAACTTCGAGGACGGAGGAGCCGTCAC
CGTCACCCAAGACACCTCCCTCGAGGACGGAACCCTCATCTACAAGGTCAAGCTCC
GTGGAACCAACTTCCCACCAGACGGACCAGTCATGCAAAAGAAGACCATGGGATGG
GAGGCCTCCACCGAGCGTCTCTACCCAGAGGACGGAGTCCTCAAGGGAGACATCAA
GATGGCCCTCCGTCTCAAGGACGGAGGACGTTACCTCGCCGACTTCAAGACCACCTA
CAAGGCCAAGAAGCCAGTCCAAATGCCAGGAGCCTACAACGTCGACCGTAAGCTCG
ACATCACCTCCCACAACGAGGACTACACCGTCGTCGAGCAATACGAGCGTTCCGAG
GGACGTCACTCCACCGGAGGAATGGACGAGCTCTACAAGTGAtttcacttccagttaatttatatatttt
gaacttatttttattcccacccgattttcagtgactgtcaaaaattcacataattcgttctttcatttttttttcttttcaagaattttcactttgaatgttttcc
gaataaaactgtacagtgtcattcattcaatgtttttcttaaaacaaacaactgaattatataaaactggtttattgtgattgtactatatatataatac
accggagtaaaataaacaggcaaattgatatgacagtttgaacgtcagcttgatttgtagagatcacaccgtttcagtaaaga

Strain names and genotype: PHX8481 *sybIs8481[Pges-1::unc-85::unc-85 3'UTR, Pmyo-2::gfp]*) and PHX8486 *sybIs8486[Pges-1::unc-85::unc-85 3'UTR, Pmyo-2::gfp]*)

Sequence of pPD49.78 (*Pges-1::unc-85::unc-85* 3'UTR) -plasmid

unc-85 promoter is labeled with green, *unc-85* exons are labeled with yellow and orange, and *unc-85* 3' UTR is labeled with gray.

ATGACCATGATTACGCCAAGCTTGCATGCgtgcttaaagagaagactgttagccactcagccacttcagccatatcgaaa
cagtcatattggctctctttcagatcacttaaaactccgaactatgatgacgaaaaaatgttttcttgaattttcat
tttattgtgaaattgaaaactctgcatagcccgcagtcacttgaaagcacatttaagatggaatcaaatggtaagaa
agctcgtgtaatcaggagattttatccggggtatgccgaggaatatatggtgcactcgcattagctggcatttttttg
taattatcggatttctaccttttttcttaatctagtttgttatcattgtccatcatttacaaccaaaaatttgcgtca
tttacaaccaaaaattgatctgacaattatcataattcggtttttattttcagaagaaacgctccgatgtgatccta
tagtgaatatacttttccatcctctctgaaataaacaattatgaagaatgcatcaatttgcattcgaaataactgcc
gtattttgtctattggtatggctgcagtaccatttttcttcgcgtctttgcgcagccttcgggcgctaccaataagg
ctaagccggggtaagttatttcagaattctaatcaactaatctaagtctcatacatcattgtcaagtgacgcatttt
tttaatttctatgttaactcgactttaatattcttttctggtttcaccagaaaaatgctggactgaaaaattgaatt
tcccgcgatttgggaaaaaccgcagtttaagcttttggcatgaatacagtgaacacaggaacaaaaaattgacttgt
tattttgagtttatattaaatgttaattttgcatctcatattttcacaaaacttgtttgcttgaaaaatgtgcaaat
aacaagtgaacatacatcaaggtttatgcagatcagcactgatgcaactgatagcaaaactgataagggtcaaaatt
tcagtggccagcacaaacacatacacattcgtagttcgttttgtgtttcgtgtatttagcatttattttcaattcc
ggcaatttgccgatttgccggaaattttgatttttggcaaattgccagtttgccgtttgccgaacatcaatttgccg
aaattttttagagggatctttataagacggaaacacttaaaattgtgctttttttggaatttttttcaatgtttcaat
agaattagcttacatttaaaattagatgtaggaacgtttataggacgcttacaattttgccgattaaaatcgaagtt
ctgaacttttcaaaaaatgtgcaaaatttttttttgacaaatttgacaaatcggccggtttgtcgattgtccggaatt
tttcagtttcggcgatttaccggtttgccggaaaacatcgtttgccacccacacacacataaagagacgaaacaaca
caccatcttcaaaaaatttgtgaatcttcatttccacgtggtttgcagggtttcccatccaccccactctatctctc
caagttgaattttggcaaaattgaccacacaccgatcaaaaactattgtgtgtgtgtgtatttgtgtcttcaataat
tcctgtctgtgtctcgcactatctattgactacaagaacgtgatcgcagctctacgaaacctttactttattttaga
aatatttttttcatgctattagcaaaaaaaaaagtttttttttgtctaagtacacttaaaatttccaaaatttttaag
atttttcaaaatgtgcgatcaaagatctgacaaaatgccaaaattttgaagaggaaatttagtcggcttccaaaatt
ataagtggcagaaattgagcaattgtcacttttttgacagtaaatagaaaattttttgaaaaattttgaaacgcttta
ttttgttacttggtcattttggcaccataggagtagtttttaacattttccccactggcgctacttcattttaaact
tttccaaagatttaatttttaattttaaaaaatttcatataattcgtctgcgtcttacagtttcaggcttcaaaaa
tctattacatatcttatctttgaattcagATGGCTTCCCGTGTCAACATTGTTCAAGTACAAATTCTCGACAATCCT
GCGATGTTTGTCGACAAATTCAAGTTGGAGATCACATTTGAAGTTTTTGAGCACTTGCCGCATGgtaagaaagagga
aaatataaactcatcgataataatgataattttcagATCTCGAATGGGAATTGGTCTACGTCGGATCCGGAACATCC
CGAGACTTTGACCAAGTTCTCGATTCAGCGCTCGTTGGTCCAATTCCAGAAGGACGCCACAAGTTTGTGTTCGACGC
GGATCATCCGGATATCTCGAAGATCCCAGTCGATGATATCGTCGGTGTCAGCGTACTTCTCCTGCGCTGCAAGTACA
ACGATCAGGAGTTTATCAATATGGGATGGTTCGTGGCAAATGAGTACACCGAGGAAGAGCTCAAAGAGAATCCCCCA
TCGCAGCCACTCATCGAAAAGCTTTCCCGCAAAGTCGAAACTGAAGATCTTCGCATCACCACATTCCCGATTCGCTG
GACTGATGAGGATCCGGTCGCAGAGCCTGTCGAGGATGAGGCTAACAGAGTATTCGCGGAAGATGACCTCATGCCTC
TGAATGATGATGGCCAGGAAGATGACGATGAGGAGGAAGAGgtgagaaaatgaattttacttattcaaaaattaaat
aattttattcagGATGATGACGAGATGGAGGCCAACGCTGAAGAAGTGGATTTGAACGAGAGCTTCAATGAACGATT
GGCCAATGCACTTGACGGGGCCGAACAGAAAGGCGCAGATGAGAAAATGGAGGACGATGGAGCAAATGAGGATGTTG
ATATGGCTGgtaagtgttacttggtttctctaaccccttcttgtttcgtgatgtgtgcttctcaccctgtaattata
gATGACGAGCCTGGCGTTCAGATCAACACTGACACGAAAGTTCCGGAATCGATGGCGGAGCCACTCTCAGACAAGAC
AAACAACGAAATGGTCCAGTGAtttcacttccagttaatttatatattttgaacttatttttattcccacccgattt
tcagtgactgtcaaaaattcacataattcgttctttcatttttttttcttttcaagaattttcactttgaatgttttc
cgaataaaactgtacagtgtcattcACTAGTCGGCCGTACGGGCCCTTTCGTCTCGCGCGTTTCGGTGATGACGGTGAAAACC
TCTGACACATGCAGCTCCCGGAGACGGTCACAGCTTGTCTGTAAGCGGATGCCGGGAGCAGACAAGCCCGTCAGGGCGCGTCAGCG
GGTGTTGGCGGGTGTCGGGGCTGGCTTAACTATGCGGCATCAGAGCAGATTGTACTGAGAGTGCACCATATGCGGTGTGAAATACC
GCACAGATGCGTAAGGAGAAAATACCGCATCAGGCGGCCTTAAGGGCCTCGTGATACGCCTATTTTTATAGGTTAATGTCATGATA
ATAATGGTTTCTTAGACGTCAGGTGGCACTTTTCGGGGAAATGTGCGCGGAACCCCTATTTGTTTATTTTTCTAAATACATTCAAA

TATGTATCCGCTCATGAGACAATAACCCTGATAAATGCTTCAATAATATTGAAAAAGGAAGAGTATGAGTATTCAACATTTCCGTG
TCGCCCTTATTCCCTTTTTTGCGGCATTTTGCCTTCCTGTTTTTGCTCACCCAGAAACGCTGGTGAAAGTAAAAGATGCTGAAGAT
CAGTTGGGTGCACGAGTGGGTTACATCGAACTGGATCTCAACAGCGGTAAGATCCTTGAGAGTTTTCGCCCCGAAGAACGTTTTCC
AATGATGAGCACTTTTAAAGTTCTGCTATGTGGCGCGGTATTATCCCGTATTGACGCCGGGCAAGAGCAACTCGGTCGCCGCATAC
ACTATTCTCAGAATGACTTGGTTGAGTACTCACCAGTCACAGAAAAGCATCTTACGGATGGCATGACAGTAAGAGAATTATGCAGT
GCTGCCATAACCATGAGTGATAACACTGCGGCCAACTTACTTCTGACAACGATCGGAGGACCGAAGGAGCTAACCGCTTTTTTGCA
CAACATGGGGGATCATGTAACTCGCCTTGATCGTTGGGAACCGGAGCTGAATGAAGCCATACCAAACGACGAGCGTGACACCACGA
TGCCTGTAGCAATGGCAACAACGTTGCGCAAACTATTAACTGGCGAACTACTTACTCTAGCTTCCCGGCAACAATTAATAGACTGG
ATGGAGGCGGATAAAGTTGCAGGACCACTTCTGCGCTCGGCCCTTCCGGCTGGCTGGTTTATTGCTGATAAATCTGGAGCCGGTGA
GCGTGGGTCTCGCGGTATCATTGCAGCACTGGGGCCAGATGGTAAGCCCTCCCGTATCGTAGTTATCTACACGACGGGGAGTCAGG
CAACTATGGATGAACGAAATAGACAGATCGCTGAGATAGGTGCCTCACTGATTAAGCATTGGTAACTGTCAGACCAAGTTTACTCA
TATATACTTTAGATTGATTTAAAACTTCATTTTTAATTTAAAAGGATCTAGGTGAAGATCCTTTTTGATAATCTCATGACCAAAAT
CCCTTAACGTGAGTTTTCGTTCCACTGAGCGTCAGACCCCGTAGAAAAGATCAAAGGATCTTCTTGAGATCCTTTTTTTCTGCGCG
TAATCTGCTGCTTGCAAACAAAAAAACCACCGCTACCAGCGGTGGTTTGTTTGCCGGATCAAGAGCTACCAACTCTTTTTCCGAAG
GTAACTGGCTTCAGCAGAGCGCAGATACCAAATACTGTCCTTCTAGTGTAGCCGTAGTTAGGCCACCACTTCAAGAACTCTGTAGC
ACCGCCTACATACCTCGCTCTGCTAATCCTGTTACCAGTGGCTGCTGCCAGTGGCGATAAGTCGTGTCTTACCGGGTTGGACTCAA
GACGATAGTTACCGGATAAGGCGCAGCGGTCGGGCTGAACGGGGGGTTCGTGCACACAGCCCAGCTTGGAGCGAACGACCTACACC
GAACTGAGATACCTACAGCGTGAGCATTGAGAAAGCGCCACGCTTCCCGAAGGGAGAAAGGCGGACAGGTATCCGGTAAGCGGCAG
GGTCGGAACAGGAGAGCGCACGAGGGAGCTTCCAGGGGGAAACGCCTGGTATCTTTATAGTCCTGTCGGGTTTCGCCACCTCTGAC
TTGAGCGTCGATTTTTGTGATGCTCGTCAGGGGGGCGGAGCCTATGGAAAAACGCCAGCAACGCGGCCTTTTTACGGTTCCTGGCC
TTTTGCTGGCCTTTTGCTCACATGTTCTTTCCTGCGTTATCCCCTGATTCTGTGGATAACCGTATTACCGCCTTTGAGTGAGCTGA
TACCGCTCGCCGCAGCCGAACGACCGAGCGCAGCGAGTCAGTGAGCGAGGAAGCGGAAGAGCGCCCAATACGCAAACCGCCTCTCC
CCGCGCGTTGGCCGATTCATTAATGCAGCTGGCACGACAGGTTTCCCGACTGGAAAGCGGGCAGTGAGCGCAACGCAATTAATGTG
AGTTAGCTCACTCATTAGGCACCCCAGGCTTTACACTTTATGCTTCCGGCTCGTATGTTGTGTGGAATTGTGAGCGGATAACAATT
TCACACAGGAAACAGCT
